# Hepatitis B reactivation is a rare event among patients with resolved infection undergoing anti-CD20 antibodies in monotherapy without antiviral prophylaxis: results from the HEBEM study

**DOI:** 10.1007/s00415-023-11973-y

**Published:** 2023-09-11

**Authors:** Blanca Marzo, Angela Vidal-Jordana, Joaquín Castilló, Miguel-Angel Robles-Sanchez, Susana Otero-Romero, Mar Tintore, Xavier Montalban, Maria Buti, Mar Riveiro-Barciela

**Affiliations:** 1 Department of Medicine, Universitat Autònoma de Barcelona (UAB), Bellaterra, Spain; 2 Liver Unit, Internal Medicine Department, Hospital Universitari Vall d’Hebron, Vall d’Hebron Barcelona Hospital Campus, Barcelona, Spain; 3 Servei de Neurologia. Centre d’Esclerosi Múltiple de Catalunya (Cemcat). Hospital Universitari Vall d’Hebron, Universitat Autònoma de Barcelona, Barcelona, Spain; 4 Grup d’Investigació Multidisciplinari d’Infermeria, Vall d’Hebron Institut de Recerca (VHIR), Vall d’Hebron Barcelona Hospital Campus, Barcelona, Spain; 5 Servei de Medicina Preventiva i Epidemiologia. Hospital Universitari Vall d’Hebron, Universitat Autònoma de Barcelona, Barcelona, Spain; 6 Centro de Investigación Biomédica en Red de Enfermedades Hepáticas y Digestivas (CIBERehd), Instituto de Salud Carlos III, Madrid, Spain

**Keywords:** Rituximab, Ocrelizumab, Hepatitis B, Reactivation, Anti-CD20 antibodies, Multiple Sclerosis

## Abstract

**Introduction:**

Prospective data on the risk of hepatitis B reactivation (HBVr) among patients with resolved HBV infection undergoing anti-CD20 antibodies monotherapy is scarce***.*** We aimed to assess the risk of HBVr in patients with resolved HBV infection treated with rituximab or ocrelizumab in monotherapy for multiple sclerosis (MS) or neuromyelitis optica spectrum disorder (NMOSD) without antiviral prophylaxis.

**Methods:**

HEBEM is a prospective study that included all consecutive adults HBsAg-negative/anti-HBc-positive who initiated anti-CD20 antibodies for MS or NMOSD at Cemcat. Inclusion criteria encompassed undetectable HBV-DNA, absence of other immunosuppressants or antiviral therapy. Every 6 months HBsAg, ALT and HBV-DNA were performed to rule out HBVr (defined by 2-log increase in HBV-DNA or seroconversion to HBsAg+).

**Results:**

From August/2019 to August/2022, 540 subjects initiated anti-CD20 antibodies, 28 (5.2%) were anti-HBc-positive and were included. Twenty-two received rituximab and 6 ocrelizumab. The majority (89.3%) had previously received ≥ 1 immunomodulatory drug, with corticosteroids (82.1%) and interferon (42.9%) as the most common. At inclusion, all presented normal transaminases and undetectable HBV-DNA. Median anti-HBs levels were 105.5 mIU/mL (IQR 0–609). Median follow-up was 3.1 years (2.1–4.0). Median number of cycles of anti-CD20 antibodies was 6 (3–7), with a cumulative dose of 8.5 g (5.8–11.2) of rituximab and 3 g (1.8–3.8) of ocrelizumab. Neither cases of HBVr nor changes in anti-HBs titers were observed per 83.6 patient-years treated with monotherapy with anti-CD20 antibodies.

**Conclusions:**

In this cohort of patients with MS or NMOSD and resolved HBV infection, anti-CD20 monotherapy was not associated with detectable risk of HBV reactivation despite the lack of antiviral prophylaxis.

## Introduction

According to the World Health Organization (WHO), 296 million people are living with chronic hepatitis B [23]. Yet, the statistics for subjects with evidence of resolved hepatitis B virus (HBV) infection is even higher, ranging from 3.5% in Western countries to more than 20% population in high-endemicity areas [17, 19]. Patients with current HBV infection are at risk of reactivation when they are exposed to immunosuppressant drugs, as well as those with resolved HBV infection, though the risk is lower. The relevance of HBV reactivation (HBVr) lies in the potential risk of developing acute hepatitis that can progress to chronic hepatitis and even liver failure with a high mortality rate [13]. The development of HBVr can also lead to a transient or persistent discontinuation of immunosuppressant drugs which might negatively impact on the overall and progression-free survival rate of patients [10]. Risk of reactivation varies according to the HBV status and the type of immunosuppressive treatment [15]. Rituximab-containing regimens account for the highest incidence of HBVr, with up to 55% of haematological patients, especially if undergoing R-CHOP, experiencing increases in HBV-DNA levels or reverse seroconversion to HBsAg in those with resolved HBV infection [11]. This fact has turned up in the accepted worldwide recommendation of antiviral prophylaxis for onco-hematological subjects on rituximab, regardless of whether they are HBsAg carriers or just anti-HBc-positive individuals [1, 20]. Chronic HBV individuals or those with occult HBV infection treated with rituximab seem to present a high incidence of HBVr even in non-haematological settings [6, 7, 25]. However, regarding patients with resolved HBV infection, later data have pointed out that the hazard associated with anti-CD20 monoclonal antibodies in non-haematological subjects may be quite low [3, 22]. For instance, a cohort including all anti-HBc-positive/HBsAg-negative subjects treated with rituximab from 2009 to 2013 reported cases of reactivation just in those with underlying lymphoma treated with polychemotherapy, without cases among other indications for rituximab [8]. Nevertheless, results from anti-HBc-positive individuals derive from retrospective studies including a limited and heterogeneous cohort of patients, mainly affected by rheumatological disorders and treated with a highly variable number and types of immunosuppressant drugs [12].

Multiple sclerosis (MS) is the most common inflammatory-demyelinating disease of the central nervous system. It has been estimated that more than 2 million people worldwide are suffering from MS or a neuromyelitis optica spectrum disorder (NMOSD) [21]. In recent years, the use of B-cell–targeted agents, mainly rituximab and ocrelizumab, to treat these neurological disorders has dramatically increased [9]. In this population, these drugs are given in monotherapy for long periods, providing a homogeneous and well-characterized population for addressing the real risk of HBVr associated with rituximab monotherapy.

The aim of our study was to assess the risk of HBVr in patients with evidence of resolved HBV infection (anti-HBc positive/HBsAg negative/undetectable HBV-DNA) treated with monotherapy with anti-CD20 antibodies (rituximab or ocrelizumab) for MS or NMOSD, in the absence of antiviral prophylaxis.

## Patients and methods

### Study design

HEBEM is an interventional prospective study that included all consecutive adults with MS or NMOSD undergoing treatment with anti-CD20 monoclonal antibodies (rituximab, ocrelizumab) with evidence of resolved HBV infection.

From August 2019 to August 2022, all patients with MS or NMOSD attended at the Centre d’Esclerosi Múltiple de Catalunya (Cemcat) were tested for viral hepatitis B markers prior to starting anti-CD20 in monotherapy. Ocrelizumab was administered as a single 600 mg IV infusion every 6 months, except for the initial 600 mg dose that is administered as two separate IV infusions of 300 mg. Rituximab was administered at a dose of 2000 mg (two courses of 1000 mg IV infusions separated by 2 weeks); usually, after the first year of treatment, if the response was considered adequate, patients were maintained with a lower dose (1000 mg every 6 months).

The HBV markers encompassed HBsAg and anti-HBc. Moreover, anti-HBs and HBV-DNA were also tested in all anti-HBc positive subjects. The inclusion criteria were patients with resolved HBV infection (defined by the presence of anti-HBc positive and HBsAg negative with or without anti-HBs and undetectable HBV-DNA) monotherapy with anti-CD20 monoclonal antibodies for MS or NMOSD [1]. Exclusion criteria encompassed concomitant use of other immunosuppressant drugs than anti-CD20 agents, including any dose of daily corticosteroids except for pulses, and antiviral prophylaxis with any nucleos(t)ide analogue, including TDF-containing regimens for human immunodeficiency virus (HIV) infection. All included patients underwent a clinical and blood test evaluation every 6 months in order to assess the risk of HBVr in this population.

HBV reactivation was defined by a 2-log increase in HBV-DNA levels or reverse seroconversion to positive HBsAg [2]. Pre-emptive antiviral therapy was to be started in all patients who met the criteria for HBVr during follow-up, regardless of the ALT values or liver function tests.

This study was approved by the Vall d’Hebron Hospital ethics committee and the Spanish Agency of Medicines and Medical Devices (Code: MMR-RIT-2019-01; Date 02/10/2019), and was conducted in compliance with the principles of the Declaration of Helsinki, Good Clinical Practice guidelines and local regulatory requirements. All included patients signed the informed consent for participation in the present study.

### Methods

Baseline characteristics of the included patients were collected and included age, gender, race, prior medical conditions and surgical history, type of MS or NMOSD and previous and current specific treatments. A blood test including HBV serologies (HBsAg, anti-HBc, anti-HBs), HBV-DNA and liver biochemistry was performed. Prospective follow-up every 6 months included analytical screening for HBVr, and clinical events such as progression of the neurological disorder, need of pulses of corticosteroids, changes in specific therapy (intermittent/permanent discontinuation and reason), development of adverse events, especially infectious complications, death and lost to follow-up. In those patients who required discontinuation of anti-CD20 therapy, screening of HBVr was continued for the next 12 months.

Serological markers for HBV (HBsAg, anti-HBc, and anti-HBs) were analysed by commercial enzyme immunoassays. The lower limit of quantification of anti-HBs was 3.10 mIU/mL and the upper limit, 1000 mIU/mL. Serum HBV-DNA was quantified by PCR (COBAS 6800 HBV test (Roche Diagnostics, Mannheim, Germany): lower limit of quantification, 20 IU/mL and lower limit of detection, 10 IU/mL.

### Statistical analysis

Normally-distributed quantitative variables were compared with the paired Student *t* test and expressed as the mean ± standard deviation (SD). Variables with a non-normal distribution were analysed with the Mann–Whitney *U* test and expressed as the median and interquartile range. Categorical variables were compared using the chi-square or Fisher exact test, as appropriate, and expressed as frequencies and percentages. *P* values < 0.05 were considered statistically significant. All analyses were carried out using Stata version 16 (from StataCorp, College ​​Station, Texas, USA).

## Results

### Characteristics of all patients treated with anti-CD20 antibodies

Overall, 540 patients with MS or NMOSD underwent anti-CD20 therapy during the period of study. The majority (73.2%) received rituximab and 144 (26.6%) ocrelizumab. Three-hundred and forty-two were women (63.3%). The mean age was 46.6 (SD 10.8) years and the main indication for anti-CD20 therapy was MS (92.9%).

All patients tested negative for HBsAg, anti-HCV and HIV. Overall, 28 subjects were anti-HBc positive, therefore the prevalence of resolved HBV infection in the cohort was 5.2%. Anti-HBc-positive subjects were older than those negative (53.9 vs. 46.2 years, *p* < 0.001). Likewise, the prevalence of anti-HBc positivity was higher among individuals aged over 50 years in contrast to those younger (9.4% vs. 2.7%, *p* = 0.001). Moreover, anti-HBc-positive patients were more frequently male, although this difference did not reach statistical significance (46.4% vs. 36.1%, *p* = 0.183). Race distribution was slightly different in patients with resolved HBV infection compared with those without evidence of prior HBV, with a higher rate of non-Caucasian population (Caucasian: 89.3% vs. 95.9%, African: 7.1% vs. 1.4%, Asian: 3.6% vs. 1.4%, Hispanic: 0% vs. 1.4%, *p* = 0.085). Preferred anti-CD20 agent did not differ in the group of anti-HBc-positive individuals in comparison with the overall cohort (rituximab: 78.6% vs. 73.0%, *p* = 0.346). The rate of subjects with baseline detectable anti-HBs titers was greater among those with resolved HBV infection in comparison with anti-HBc-negative subjects (75.0% vs. 23.9%, *p* < 0.001).

### Baseline characteristics of patients with resolved HBV infection treated with anti-CD20 antibodies without antiviral prophylaxis

Baseline characteristics of the 28 anti-HBc-positive individuals are summarized in Table 1. Briefly, 15 (53.6%) were female, median age 55 years and mainly Caucasian (89.3%). All patients suffered from MS except two with NMOSD. Twenty-two (78.6%) subjects with resolved HBV infection received therapy with rituximab and 6 (21.4%) ocrelizumab.

**Table 1:** Baseline characteristics of subjects with resolved HBV infection treated with anti-CD20 monoclonal antibodies

	Anti-HBc-positive subjects *N* = 28
Female gender	15 (53.6%)
Age, years	55.0 (49.7–57.5)
Ethnicity	
Caucasian	25 (89.3%)
African	2 (7.1%)
Asian	1 (3.6%)
Tobacco use	10 (34.5%)
Demyelinating disease	
SPMS	13 (46.4%)
RRMS	8 (28.6%)
PPMS	5 (17.9%)
NMOSD	2 (7.2%)
Comorbidities	
Arterial hypertension	8 (27.6%)
Dyslipidemia	6 (20.7%)
Diabetes mellitus	2 (6.9%)
Depressive disorder	14 (48.3%)
Prior immunomodulatory therapy	27 (96.4%)
Prior need of corticosteroids pulses	23 (82.1%)
Number of previous pulses	6 (4–13)
Time (months) from diagnosis of MS/ NMOSD to anti-CD20 therapy	10.5 (5.5–18.0)
Current anti-CD20 agent	
Rituximab	22 (78.6%)
Ocrelizumab	6 (21.4%)
Lymphocytes count, ×10E9/L	1.95 (1.60–2.38)
Platetets, ×10E9/L	245 (211–288)
AST, IU/mL	21 (17–25)
ALT, IU/mL	20 (15–27)
GGT, IU/mL	22 (15–59)
Anti-HBs >10mIU/mL	21 (75.0%)
Anti-HBs, mIU/L	105.5 (0–609.3)

Variables are expressed as *n* (%) or median (IQR)

*MS* multiple sclerosis, *NMOSD* neuromyelitis optica spectrum disorder, *PPMS* primary progressive multiple sclerosis, *RRMS* remittent-recurrent multiple sclerosis, *SPMS* secondary progressive multiple sclerosis, *AST* aspartate aminotransferase, *ALT* alanine aminotransferase, *GGT* gamma-gluamyltranferase.

**Fig. 1:**
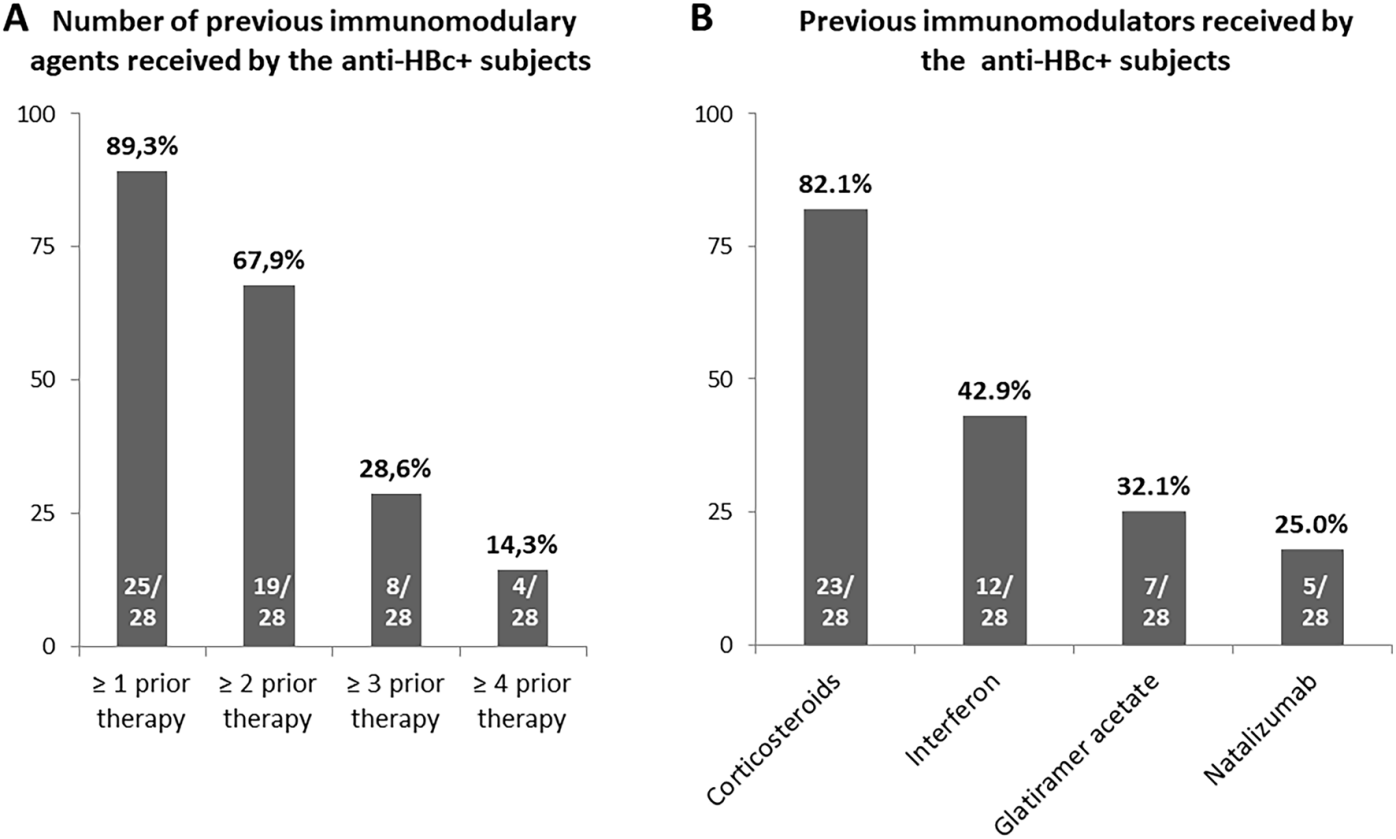
Previous immunomodulatory drugs received by patients with resolved HBV infection. **A** Number of prior immunomodulatory drugs. **B** Type of prior immunomodulatory drugs received by subjects with resolved HBV infection

The majority of anti-HBc-positive subjects (96.4%) had been previously treated with at least another immunomodulatory drug prior to the anti-CD20 therapy, as summarized in Fig. 1A. The prior most common drugs were corticosteroids pulses and interferon (Fig. 1B). Median time between the diagnosis of the neurological disease and the beginning of anti-CD20 therapy was 10.5 months. At the beginning of either rituximab or ocrelizumab, all patients presented normal aminotransferase levels and, according to the study protocol, in all cases, HBV-DNA was undetectable at inclusion. Median baseline anti-HBs titers were 105.5 mUI/mL, with all patients but 7 presenting titers over the 10 mIU/mL cut-off.

### Hepatitis B reactivation and clinical outcomes of anti-HBc-positive patients

**Fig. 2:**
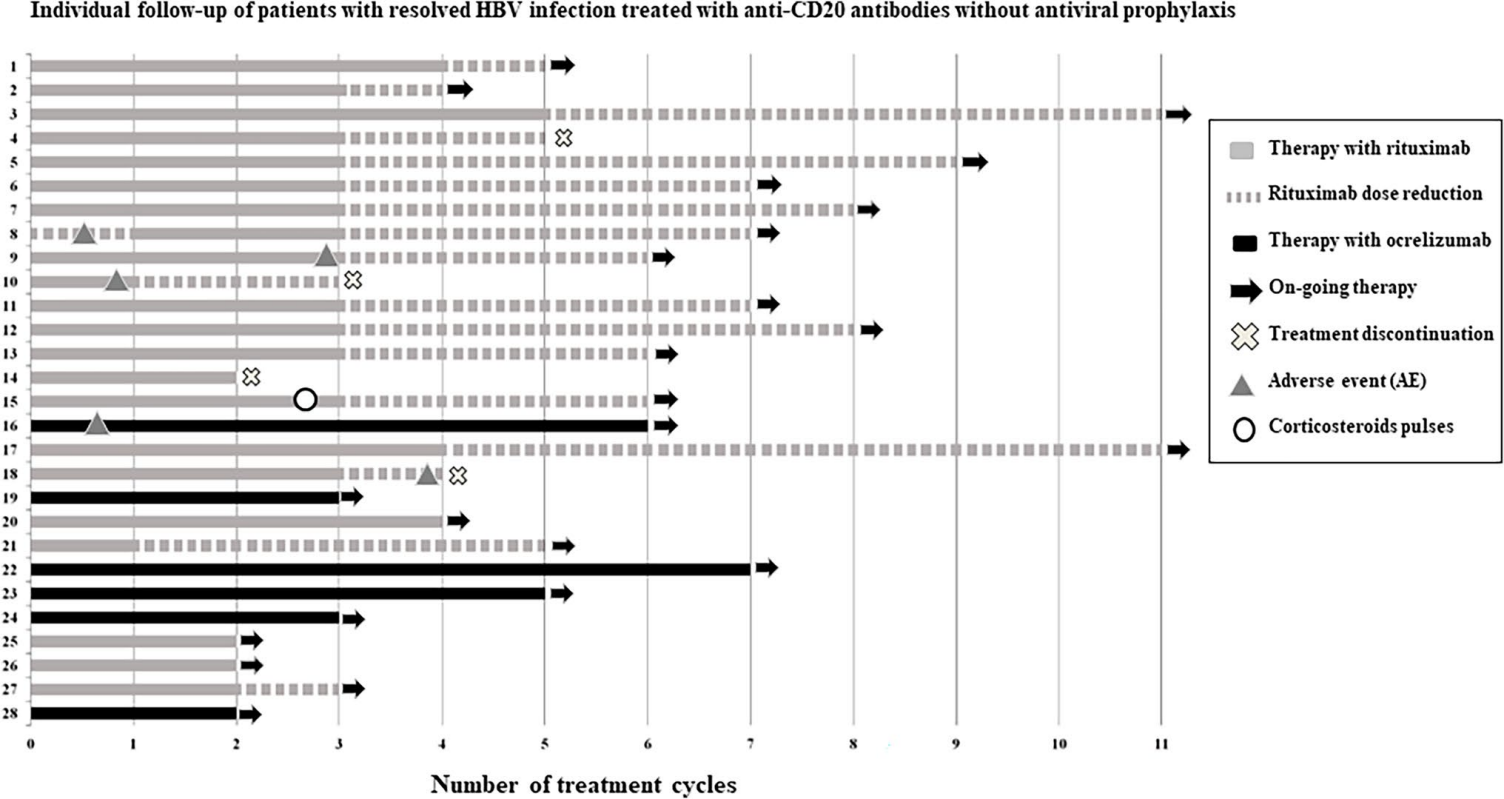
Individual follow-up of patients with resolved HBV infection treated with anti-CD20 antibodies without antiviral prophylaxis

Median follow-up of the cohort of anti-HBc-positive subjects was 3.1 years (IQR 2.1–4.0) and the median number of cycles of anti-CD20 antibodies 6 (3–7) cycles, with a median cumulative dose of 8.5 g (IQR 5.8–11.2) of rituximab and 3 g (IQR 1.8–3.8) of ocrelizumab. No cases of HBV reactivation were observed despite the lack of antiviral prophylaxis in this cohort including 83.6 patient-years at risk. The individual follow-up of anti-HBc-positive subjects is shown in Fig. 2.

One subject included in this study received a 3-dose corticosteroid pulse (dose of 1 gr per day) due to a potential relapse of the disease (myelitis). Among the 22 patients treated with rituximab, dose adjustment was carried out in 17 (77.3%) individuals, the majority according to protocol due to acceptable disease control, and in 2 patients due to recurrent infections (one ophthalmic herpes during the first cycle of rituximab; one due to recurrent urinary infections) and in one because of colitis as adverse event (Fig. 2).

Overall, anti-CD20 therapy was permanently discontinued in 4 (14.3%): 1 due to pregnancy aspiration, 1 by decision of the patient during the SARS CoV-2 pandemic, 1 due to recurrent urinary infections despite previous dose adjustment and 1 because of adverse event (colitis). Main outcomes and events associated with anti-CD20 are shown in Table 2. During the period of study, no changes in anti-HBs titers were observed, including the rate of subjects with anti-HBs over 10 mIU/mL (Table 3).

**Table 2:** Clinical events in the cohort of subjects with resolved HBV infection treated with anti-CD20 monoclonal antibodies without antiviral prophylaxis

	Anti-HBc-positive individuals *N* = 28
Anti-CD20 therapy duration	
Months of therapy	37.9 (25.6–48.5)
Number of cycles	6 (3–7)
HBV reactivation	0 (0%)
Need of corticosteroids pulses	1 (3.6%)
Infectious complications	3 (10.7%)
Permanent discontinuation of therapy	4 (14.3%)
Rituximab dose adjustment due to infections/side effects (*N* = 22)	3 (13.6%)

Variables are expressed as *n* (%) or median (IQR)

**Table 3:** Evolution of anti-HBs titters in the cohort of subjects with resolved HBV infection treated with anti-CD20 agents without antiviral prophylaxis

	Baseline	Last follow-up	*p* value
Anti-HBs (mUI/mL) titers	105.5 (0–609.3)	92 (13–493.5)	0.502
Anti-HBs > 10mUI/mL	21 (75.0%)	22 (78.6%)	0.749

Variables are expressed as *n* (%) or median (IQR)

## Discussion

Herein, we present data from a prospective study that follows 83.6 patient-years with resolved HBV infection treated with anti-CD20 monoclonal antibodies in monotherapy, and no cases of HBV reactivation were observed, despite the lack of antiviral prophylaxis.

The widespread use of rituximab and other anti-CD20 in multiple diseases, mainly autoimmune disorders such as rheumatoid arthritis, systemic lupus erythematosus and MS, has raised the question about the risk of HBVr linked with these drugs in non-hematological settings. These patients have a different profile, with a not so considerable degree of immunosuppression, and in most of the cases, with the anti-CD20 treatment being used as monotherapy.

The progressive and profound B-cell depletion produced by the repeated treatment with anti-CD20 drugs is the supposed mechanism associated with HBVr [24]. However, control of HBV infection is mainly driven by memory T-cell immunity [4], whose response is not so altered by these drugs [18].

In order to assess the real risk of HBVr, patients with resolved HBV infection undergoing anti-CD20 agent to treat MS or NMOSD are an excellent group of study: this population usually receive either rituximab or ocrelizumab in monotherapy; the high efficacy of the drug precludes the use of corticosteroids as shown in our cohort where only one subject developed a relapse of the neurological disorder requiring methylprednisolone pulses; and the underlying degree of immunosuppression is low since the previous use of immunomodulatory drugs is frequent whereas prior immunosuppression is rare. Moreover, inclusion criteria for entering our study encompassed a baseline undetectable HBV-DNA in order to avoid the risk linked to the occult hepatitis B state. Our results are especially relevant in view of the low rate of screening for HBVr in patients with MS undergoing rituximab-containing regimens. A recently published multicentre study including 53 anti-HBc-positive subjects showed that 21 received antiviral prophylaxis, and only 13 had HBV DNA monitoring during therapy for assessment of HBVr [5].

In view of our results, in patients with resolved HBV infection and MS or NMOSD treated with anti-CD20 antibodies, we recommend monitoring for HBsAg and HBV-DNA every 6 months and HBV therapy only in case of evidence of reactivation.

Our study has some limitations. Firstly, the relatively limited number of subjects included, since this was a unicentric study. However, the CEMCAT is the reference center in all Catalonia for patients with MS and NMOSD leading to more than 500 subjects treated with anti-CD20 antibodies during the 3-year period of study, including 28 with resolved HBV infection who were recruited for our study. Despite the fact the follow-up of the included subjects was three years, which is a relatively short period of time considering some infections associated with rituximab [16], patients were exposed to repeated doses of the drugs and based on data from hematological cohorts, risk of HBVr is especially relevant after the second anti-CD20 exposure [14].

The strengths of our study are that this is the first prospective study in a very homogeneous group of patients with resolved HBV infection undergoing anti-CD20 monotherapy, without HBV prophylaxis. Another merit was the centralized diagnosis and follow-up of patients at the CEMCAT unit, and the periodical 6-month supervision by the Liver Unit in order to ensure proper ruling out of HBV reactivation.

In summary, in our cohort anti-CD20 monoclonal treatment used in monotherapy was not associated with risk of HBV reactivation in subjects with resolved HBV infection and multiple sclerosis or diseases of the spectrum of optic neuromyelitis at medium term follow-up, despite the lack of antiviral prophylaxis. Our results suggest the possibility of pre-emptive management of HBV reactivation in these patients, although our findings need to be confirmed in a multicentric cohort including more patients and a longer follow-up.

## Data Availability

The data that support the findings of this study are available from the corresponding author upon request.
